# Comparison of tyrosine kinase inhibitors in the treatment of metastatic renal cell carcinoma with rhabdoid and sarcomatoid differentiations

**DOI:** 10.1002/cam4.6081

**Published:** 2023-06-16

**Authors:** Kun Wang, Pengqiang Duan, Xusheng Chen, Qing Yang, Guowei Feng, Lei Diao, Zhenting Zhang, Xin Yao

**Affiliations:** ^1^ Key Laboratory of Cancer Prevention and Therapy, Department of Geniturinary Oncology, Tianjin Medical University Cancer Institute and Hospital, National Clinical Research Center for Cancer Tianjin's Clinical Research Center for Cancer Tianjin China

**Keywords:** metastasis, renal cell carcinoma, rhabdoid and sarcomatoid differentiation, tyrosine kinase inhibitors

## Abstract

**Objective:**

To investigate the efficacy of tyrosine kinase inhibitors (TKIs) in the treatment of metastatic renal cell carcinoma (mRCC) with rhabdoid (mRCC‐R) and sarcomatoid (mRCC‐S) differentiations.

**Materials and Methods:**

In this single‐institutional cohort study, we included patients with RCC with rhabdoid (RCC‐R) and sarcomatoid (RCC‐S) differentiation, who were treated with TKIs after metastasis at our institute from 2013 to 2021. Patient characteristics, treatments, and clinical outcomes were recorded and analyzed.

**Results:**

We identified 111 patients with RCC‐R or RCC‐S differentiations, of which 23 patients were included in the final analysis. Of the 23 patients, 10 (43.5%) were grouped as mRCC‐R and 13 (56.5%) as mRCC‐S. At a median follow‐up of 40 months, mRCC‐R and mRCC‐S progressed in 7 of 10 and 12 of 13 patients, respectively. In addition, four and eight patients died in the mRCC‐R and mRCC‐S groups, respectively. The median progression‐free survival (PFS) of the two groups was 19 months (mRCC‐R: 95% confidence interval [CI] 4.08–33.92) and 7 months (mRCC‐S: 95% CI 2.03–11.96), while the median overall survival (OS) was 32 months and 21 months, respectively. mRCC‐S had a worse prognosis than mRCC‐R. Based on the univariate Cox regression model, single metastasis or multiple metastasis of tumor, rhabdoid differentiation, and sarcomatoid differentiation were predictors of PFS but not OS.

**Conclusion:**

The efficacy of TKIs in the treatment of mRCC‐R and mRCC‐S may be different.

## 1 INTRODUCTION

Renal cell carcinoma (RCC) is the most common tumor of the kidney, accounting for 80% of all renal tumors.^1^, ^2^ Early RCC is mainly treated surgically. However, approximately 30% of patients present with tumor metastasis before surgery, while 30% relapse after complete resection of the primary tumor. In many patients, although the tumors are accidentally found and completely resected, they still recured.^3^ Gokden^4^ was the first to report rhabdoid differentiated RCC, which has high malignancy and poor prognosis. Rhabdoid characteristics are frequently seen in clear cell RCC, the most prevalent histopathological subtype of RCC,^5^ and they can be detected in other RCC histological subtypes as well. Sarcomatoid differentiated RCC is another histological variant that is common in rhabdoid differentiation.^2^ RCC with both rhabdoid and sarcomatoid differentiation accounts for 10%–15% of RCC cases, and the majority of patients with S/R RCC develop metastatic disease. In general, rhabdoid and sarcomatoid differentiations are rare pathological types with a high degree of malignancy. They are prone to distant metastasis during the early stages and are resistant to many conventional drugs.^6^

Oral multitarget medications known as antiangiogenic tyrosine kinase inhibitors (TKIs) target vascular endothelial growth factor (VEGF) receptors, platelet‐derived growth factor receptors, and other tyrosine kinases.^7^ For a long time, TKIs were the first‐line treatment option for metastatic RCC (mRCC). Updated clinical guidelines continue to advocate the sequential application of various antiangiogenic targeted medicines as the second and subsequent lines of therapy for mRCC, despite the use of immune checkpoint inhibitors (ICIs) having raised concerns in recent years.^8^ Of the TKIs, sunitinib and pazopanib are the first‐line drugs of choice for the treatment of mRCC. A phase III trial conducted by Sternberg et al.^9^ evaluated the efficacy and safety of pazopanib and found that progression‐free survival (PFS) and tumor response significantly increased after pazopanib treatment in treatment‐naive and cytokine‐pretreated patients with advanced and/or mRCC compared to those in placebo‐controlled patients. Another phase III trial comprising 750 patients with mRCC found that the median PFS and the objective response of the sunitinib group were better than those of the interferon‐alpha group (median PFS: 11 months vs. 5 months; objective response rate: 30% vs. 6%, *p* < 0.001).^10^ The latest guidelines show that for patients with low‐risk mRCC, the first‐line treatment is sunitinib and pezopanib, while for patients with medium‐ and high‐risk mRCC, the first‐line treatment is sunitinib/pezopanib or pabolizumab/nivolumab combined with TKI.

In clinical applications, immunotherapy has some side effects. The most common adverse reactions to anti‐PD‐1 (programmed cell death protein 1) antibody monotherapy include exertion, pruritus, nausea, and diarrhea. Adverse reactions to the combination of anti‐PD‐1 and anti‐CTLA‐4 often lead to an increase in lipase, amylase, and alanine aminotransferase activities. Anti‐PD‐1 drugs combined with anti‐VEGF drugs may cause diarrhea and hypertension. Clinically, immunotherapy in combination with targeted therapy is not suitable for all patients. Therefore, targeted TKI therapy alone is still therapeutically significant.

In view of the clinicopathological characteristics of mRCC with rhabdoid and/or sarcomatoid differentiations, we collected data on patients with RCC at the Tianjin Medical University Cancer Hospital between 2013 and 2021, with the aim of investigating whether the therapeutic effects of TKIs in mRCC‐R and mRCC‐S are different. In addition, we sought to elucidate the reasons for the differences and determine individualized treatment plans for patients with different types of tumors.

## 2 METHODS

### 2.1 Patients and treatment regimens

For the cohort study, we gathered clinical and pathological information from 6500 patients with RCC who underwent surgery at Tianjin Medical University Cancer Hospital between 2013 and 2021. Of the 6500 patients, 36 presented with RCC‐R and 75 with RCC‐S. After excluding cases with interrupted/halted follow‐ups and incomplete basic information, 26 patients with RCC‐R and 44 patients with RCC‐S were included in the final analysis.

Pathological diagnosis was based on the 2016 World Health Organization classification of renal tumors. Following radical nephrectomy, patients regularly went for check‐ups. TKIs treatments were administered after imaging examination showed metastasis. For patients with distant metastasis before the nephrectomy, TKIs were used after the patients recovered. Patient medication included the following: sorafenib, 400 mg twice daily, 4 weeks as a cycle; sunitinib, 50 mg once daily, 6 weeks as a cycle; axitinib, 5 mg twice daily, 4 weeks as a cycle; fruquintinib, 5 mg once daily, 4 weeks as a cycle; pazopanib, 800 mg once daily, 4 weeks as a cycle. The drug treatment was continued until it no longer produced clinical efficacy or until patients could no longer tolerate drug toxicity. Adverse reactions were evaluated after each cycle of treatment, and imaging examinations were performed every two cycles to evaluate the curative effect. Overall, 11 patients with RCC‐R and 22 patients with RCC‐S had metastasis. The medication after metastasis was unclear for one patient in the mRCC‐R group and for five patients in the mRCC‐S group. In addition, in the mRCC‐S group, two patients used traditional Chinese medicines and two used interferon‐α.

Overall, the inclusion criteria for the patients were as follows: RCC with rhabdoid or sarcomatoid differentiation and postoperative metastasis, use of TKI agents after metastasis, and complete basic clinical data. The exclusion criteria were as follows: RCC without rhabdoid or sarcomatoid differentiation, no metastasis after operation, no use of TKI agents after metastasis, and incomplete basic clinical data.

**FIGURE 1:**
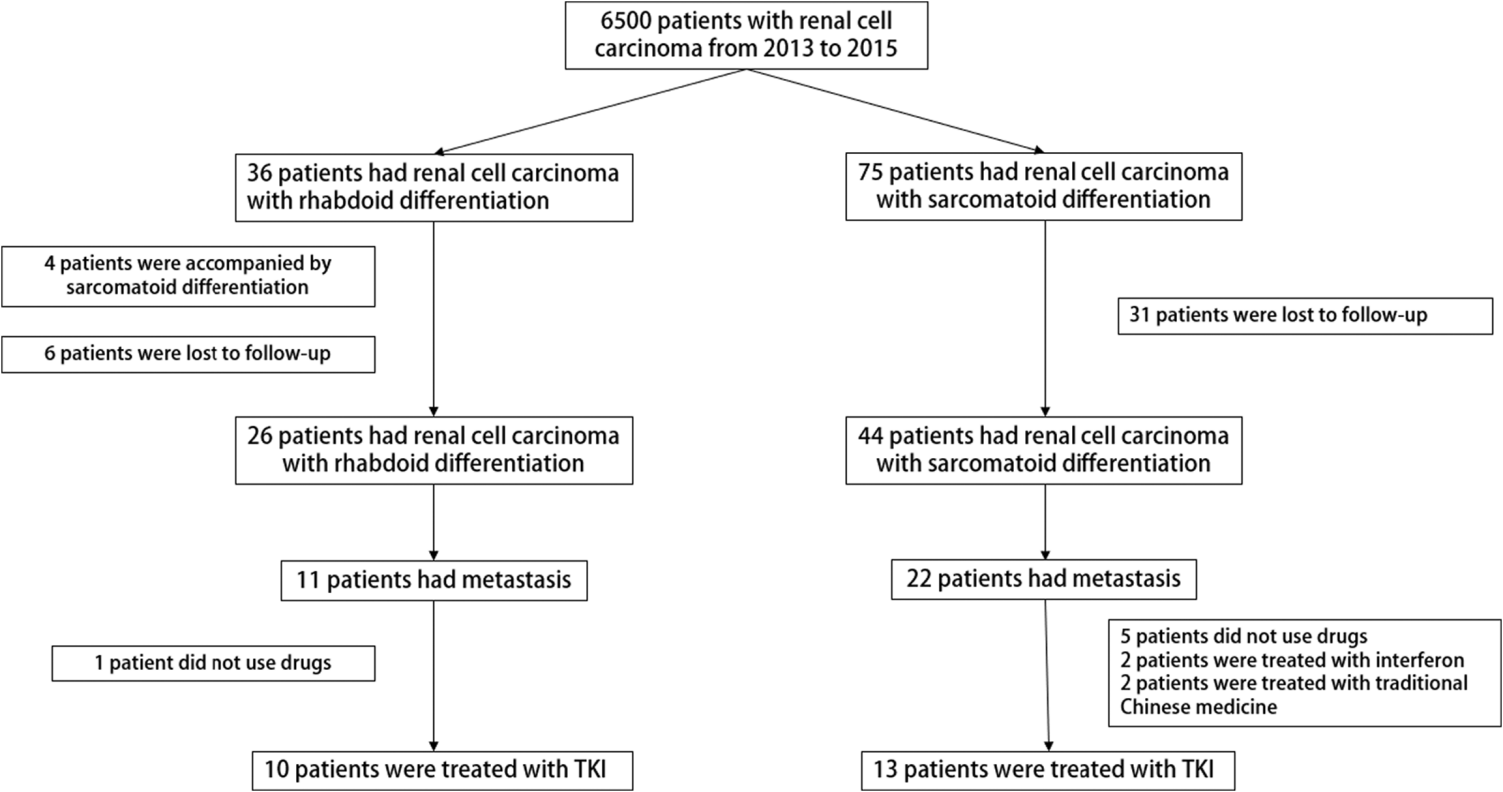
Patients included in the study. TKI, tyrosine kinase inhibitor.

Finally, 10 cases of mRCC‐R and 13 cases of mRCC‐S were included in the analysis (Figure 1). Data of the two groups were collected and analyzed, including age, sex, tumor site, size, position (left or right), T stage, N stage at the time of surgery, position, and number of tumor metastases. Telephone follow‐up and the hospital system were used to determine the time of postoperative metastasis, treatment, and prognosis.

### 2.2 Study endpoints

The primary endpoint of the study was PFS and the secondary endpoint was overall survival (OS). PFS constituted the time interval between the beginning of the treatment (after metastasis) and disease progression, intolerable adverse reactions, or death from any cause. OS constituted the time interval between the beginning of the treatment (after metastasis) and death or the end of observation.

### 2.3 Statistical analysis

SPSS v24 software (IBM Corp.) was used to conduct statistical analyses. The patient characteristics for each cohort are presented using the mean and standard deviation for continuous variables and numbers and relative percentages for categorical variables. For categorical and continuous variables, respectively, comparisons were performed using the chi‐squared test and independent samples *t*‐test.

The survival results are presented using Kaplan–Meier estimates. The median PFS results were reported at a 95% confidence interval (CI). First, we used Kaplan–Meier estimates to explore the relationship between different tumor stages and tumor metastasis in the RCC‐R and RCC‐S groups, respectively. Then, we conducted univariate Cox regression analysis. For this analysis, the patients' sex, age, primary tumor location, tumor size, T stage, metastatic site, and the presence of preoperative metastasis were considered. Age and tumor size were continuous variables. In follow‐up tests, the factors that met the linear condition of the regression analysis model were included in the model in the form of an original variable. Multiple categorical variables were used as dummy variables in the analysis. Statistical significance was set at *p* < 0.05.

## 3 RESULTS

### 3.1 Tumor staging and metastasis

**FIGURE 2:**
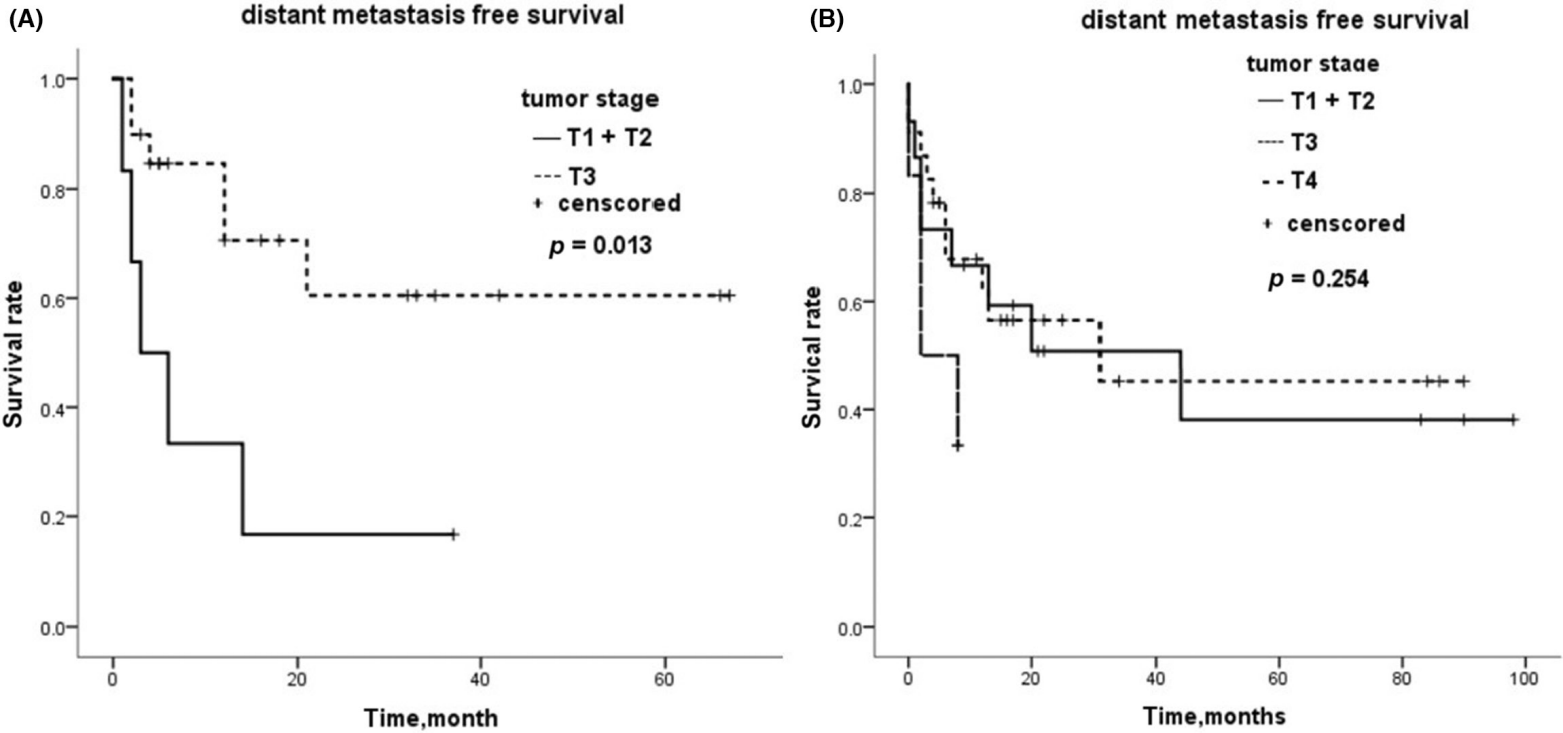
Kaplan–Meier curve for distant metastasis free survival of patients who has (A) RCC‐R and (B) RCC‐S. RCC‐R, renal cell carcinoma with rhabdoid differentiation; RCC‐S, renal cell carcinoma with sarcomatoid differentiation.

The metastasis‐free survival time for 26 patients with RCC‐R and 44 patients with RCC‐S was determined through telephone follow‐up and access to the hospital system. Kaplan–Meier estimates were performed to visualize the relationship between different tumor stages and metastasis. The tumor T stage levels included T1, T2, T3, and T4. T1 and T2 stages were combined for the analysis based on the smaller number of patients and similarity in clinical characteristics (Table S1). The results of the Kaplan–Meier survival analysis indicate that there was a significant difference between tumor stage and metastasis in the RCC‐R group (Figure 2A, *p* = 0.013). However, the distant metastasis‐free survival of patients with RCC‐R in the T3 stage was better than that of patients in the T1 and T2 stages. In contrast, no significant difference between tumor stage and metastasis was observed in the RCC‐S group (Figure 2B, *p* = 0.254). Nevertheless, the metastasis‐free survival time of patients with RCC‐S in the T4 stage was significantly worse than that of patients in the other stages.

### 3.2 mRCC‐R/mRCC‐S and prognosis after TKI treatment

Twenty‐three patients were included in this study. They were treated with TKIs after RCC relapse. Baseline data between 111 RCC (36 cases of RCC‐R and 75 cases of RCC‐S) and 23 mRCC (10 cases of mRCC‐R and 13 cases of mRCC‐S) cases were not statistically different (Table S2). This signifies that there was no selection bias due to the exclusion of patients with incomplete data. Of this subset, 10 patients were included in the mRCC‐R group and 13 in the mRCC‐S group. The patient characteristics are presented in Table 1. Most of the patients were men. All patients in the mRCC‐R group had clear cell carcinomas. Twelve patients in the mRCC‐S group had renal clear cell carcinomas, while one presented with an Xp11 22 translocation‐associated RCC. In the mRCC‐R group, seven patients had stage 3a, while one patient each had stage 1a, 1b, and 2a. In the mRCC‐S group, four patients had stage 3a, three patients had stage 1b, three patients had stage 4, two patients had stage 2a, and one patient had stage 2b. In general, most patients had stage 3a tumors. Because the number of patients with 1a and 2b tumors was too low, T1 (1a + 1b) and T2 (2a + 2b) patients were grouped for subsequent analysis. One patient in the mRCC‐R group and two patients in the mRCC‐S group had metastasis before surgery (10% and 15.4%, respectively, Table 1).

**TABLE 1:** Baseline characters of patients who has mRCC‐R and mRCC‐S.

	mRCC‐R	mRCC‐S	*p* value
Age, years	57.50 ± 7.01	59.00 ± 6.98	0.615
Man, *n* (%)	7 (70.0%)	12 (92.3%)	0.281
Tumor site (left), *n* (%)	6 (60.0%)	6 (46.2%)	0.680
Tumor size, cm	7.86 ± 2.56	7.35 ± 3.11	0.677
Tumor T stage, *n* (%)			0.261
T1 + T2	4 (40.0%)	6 (46.2%)	
T3	6 (60.0%)	4 (30.8%)	
T4	0	3 (23.1%)	
Tumor N stage, *n* (%)			0.229
N0 or Nx	10 (100%)	10 (76.9%)	
N1	0	3 (23.1%)	

*Note*: T1, tumor ≤7 cm in greatest dimension, limited to the kidney; T2, tumor >7 cm in greatest dimension, limited to kidney; T3, tumor extends into major veins or peripheric tissues, but not into the ipsilateral adrenal gland and not beyond the Gerola fascia; T4, tumor invades beyond the Gerota fascia (including contiguous extension into the ipsilateral adrenal gland).

Abbreviations: mRCC‐R, metastatic renal cell carcinoma with rhabdoid differentiation; mRCC‐S, metastatic renal cell carcinoma with sarcomatoid differentiation.

The conditions of metastasis and treatment are summarized in Table 2. At a median follow‐up time of 40 months, in the mRCC‐R group, seven patients (70%) progressed and four passed away (40%). In addition, seven patients had simple lung metastasis, one patient had lung and pleural metastasis, and the other two had bone, diaphragm, pleural, and lymph node metastases. In the mRCC‐S group, 12 patients (92.3%) progressed and eight passed away (61.5%). Regarding the transfer situation, three patients had simple lung metastasis, two patients had simple bone metastasis, one person each had simple brain and retroperitoneal lymph node metastasis, and six patients had lung metastases with other organs/regions (liver, brain, bone, peritoneum, lymph nodes, and perirenal artery). As for the use of TKIs, in the mRCC‐R group, six patients were treated with sorafenib, two were treated with axitinib, and one patient each was treated with fruquintinib and pazopanib. In the mRCC‐S group, six patients were treated with sorafenib, three patients each with sunitinib and axitinib, and one with pazopanib after metastasis (Table 2). There were no statistically significant differences in the basic clinical traits between the two groups.

**TABLE 2:** Metastasis and treatment conditions of patients who has mRCC‐R and mRCC‐S.

	mRCC‐R	mRCC‐S
Medication within 3 months after surgery, *n* (%)	4 (40.0%)	6 (46.2%)
Preoperative metastasis, *n* (%)	1 (10.0%)	2 (15.4%)
Medication, *n* (%)		
Sorafenib	5 (50.0%)	6 (46.2%)
Axitinib	2 (20.0%)	3 (23.1%)
Sunitinib	0	3 (23.1%)
Other	3 (30.0%)	1 (7.7%)
Survival, *n* (%)	7 (70.0%)	12 (92.3%)
Progression, *n* (%)	4 (40.0%)	8 (61.5%)
Median PFS, months (95% CI)	19 (4.08–33.90)	7 (2.04–11.96)
Median OS, *n* (%)	32	21

Abbreviations: mRCC‐R, metastatic renal cell carcinoma with rhabdoid differentiation; mRCC‐S, metastatic renal cell carcinoma with sarcomatoid differentiation; OS, overall survival; PFS, progression‐free survival.

The median PFS times of the mRCC‐R and mRCC‐S groups were 19 months (95% CI: 4.08–33.9) and 7 months (95% CI: 2.04–11.96), respectively. The mRCC‐R and mRCC‐S groups had median OS times of 32 and 21 months, respectively. In the univariate Cox regression model, the presence of single or multiple metastases was related to PFS (*p* = 0.016, hazard ratio [HR] 4.00, 95% CI 1.30–12.32). Although rhabdoid or sarcomatoid pathology was related to PFS, the association was not statistically significant. (*p* = 0.055, HR 2.66, 95% CI 0.98–7.24). In the regression analysis for OS, no statistically significant parameters were found (Table 3).

**TABLE 3:** Univariable analysis of predictors of PFS in patients who has metastasis renal cell carcinoma with rhabdoid or sarcomatoid differentiation.

	PFS		OS	
	HR	*p* value	HR	*p* value
Sex	2.003 (0.644–6.233)	0.230	0.299 (0.073–1.232)	0.095
Age	0.957 (0.889–1.030)	0.238	0.969 (0.890–1.054)	0.460
Tumor site	1.090 (0.431–2.759)	0.856	0.945 (0.301–2.968)	0.923
Tumor size	0.894 (0.735–1.088)	0.264	1.079 (0.886–1.315)	0.451
Tumor T stage				
T3 vs. T1 + T2	0.547 (0.195–1.534)	0.251	1.254 (0.377–4.173)	0.712
T4 vs. T1 + T2	1.600 (0.425–6.026)	0.488	0.809 (0.093–7.048)	0.848
Number of transferred parts^a^	3.997 (1.297–12.317)	0.016	1.575 (0.493–5.027)	0.443
Preoperative metastasis	1.456 (0.405–5.239)	0.565	0.035 (0–18.828)	0.296
R/S differentiation	2.660 (0.978–7.239)	0.055	1.764 (0.528–5.896)	0.357

Abbreviations: HR, hazard ratio; OS, overall survival; PFS, progression‐free survival; R/S, rhabdoid/sarcomatoid.

^a^ Number of transferred parts: group according to whether the transfer position is single or multiple.

In the mRCC‐R group, four patients underwent immunotherapy with TKIs. Of these, two patients took axitinib with sintilimab and one patient each took sorafenib with toripalimab and fruquintinib with sintilimab. In the mRCC‐S group, three patients received immunotherapy with TKIs. These patients took axitinib with sintilimab. The median PFS for patients using TKIs alone was 6 months (95% CI: 0–12.28), while that for patients using TKIs combined with immunotherapy was 10 months (95% CI: 7.43–12.57).

## 4 DISCUSSION

Both mRCC‐R and mRCC‐S have poor patient prognosis and are often associated with identifiable cancerous components. RCC with rhabdoid or sarcomatoid differentiation was defined as ISUP4 by the International Society of Urological Pathology (ISUP).^11^ These conditions exhibit poor differentiation rather than independent tissue subtypes.^12^ Interestingly, the origin and adverse prognosis of mRCC‐R and mRCC‐S are similar. Bakouny et al.^6^ and Debien et al.^13^ found that the poor prognosis of mRCC‐R and mRCC‐S may be related to their unique molecular characteristics, which involve the expression of BAP1, TP53, CDKN2A, and MYC. In particular, the overexpression of PD‐1/PD‐L1 and the epithelial–mesenchymal transition have received increasing attention.^14^, ^15^ According to the literature, RCC‐R has a higher proportion of neutrophilic inflammation, whereas RCC‐S has a higher degree of dedifferentiation and peripheral nerve infiltration.^14^ Owing to this, we believe that there are various differences between rhabdoid and sarcomatoid differentiation that need to be further explored.

Antiangiogenic VEGF‐TKIs have been used as a first‐line treatment option for RCC over the past 20 years and can greatly improve PFS and OS in patients with locally advanced or distant metastases.^16^ Kats‐Ugurlu et al.^17^ found that aggressive rhabdoid components in RCC might lead to greater expression of VEGFA, suggesting that the tumor's histological traits may be correlated with how well antiangiogenic therapy works. A study by Juloori et al.,^18^ which included 176 patients with RCC with brain metastasis, showed that the median OS of patients receiving TKIs was significantly prolonged compared to that of other patients (16.8 vs. 7.3 months, *p* < 0.001). The overall response rate for targeted therapy with the sarcomatoid component was approximately 11.1%–19%, with progression within 1 year in 33%–57% of patients,^15^ which is slightly lower than our results (nine progressed within 1 year, 69%).

Our findings suggest that the efficacy of TKIs in the treatment of mRCC‐R was better than that in mRCC‐S. Under the same application of TKIs, PFS and OS of patients with rhabdoid pathology were better than those of patients with sarcomatoid differentiation. The mRCC‐R group had a progression rate of 70% with a mortality rate of 40%, and the mRCC‐S group had a progression rate of 92.3% with a mortality rate of 61.5%. The efficacy of pazopanib was better than that of sorafenib reported in the literature.^19^, ^20^ In this study, patients on pazopanib had shorter PFS and OS than patients on sorafenib. This may be because of the low number of cases. In the mRCC‐R and mRCC‐S groups, only one patient each received pazopanib. Furthermore, recent studies suggest that resistance induced by long‐term sunitinib treatment may be associated with the activation of AXL and MET,^21^ which contributes to the overcoming of resistance. Erlotinib may be effective in the treatment of RCC‐R with INI‐1 inactivation.^22^ Alevizakos et al.^23^ reported that among the 879 patients with mRCC‐S, advanced stage and older age were independent influencing factors of poor disease‐specific survival. Because of the small sample size of the two groups of patients, we only carried out univariate Cox regression analysis. The results showed that the number of metastatic sites and the two different pathologies, rhabdoid or sarcomatoid differentiations, were related to PFS. In the future, it is necessary to include a larger sample size for multivariate Cox regression analysis.

The T stage of the tumor is closely related to prognosis. Generally speaking, the higher the stage, the worse the prognosis and the more likely it is to metastasize. Our results show that 11 patients (42.3%) had metastasis in the RCC‐R group, and 22 patients (50.0%) had metastasis in the RCC‐S group. The prognosis among different T stages was statistically different in the mRCC‐R group. However, patients with more advanced/higher stages have metastasis later than patients with lower stages. We speculate that this may be because of the small number of patients with T1 and T2 stage tumors. In the mRCC‐S group, although no statistical difference was observed, metastasis occurred significantly earlier in patients with T4 stage tumors. Overall, the prognosis of patients with mRCC‐R was better than that of patients with mRCC‐S.

In recent years, the emergence of ICIs has greatly improved the prognosis of metastatic RCC. A retrospective chart review compared the prognosis of 35 patients with metastatic RCC with a sarcomatoid component, in which 10 patients were treated with ICIs and 25 were administered other therapies. They showed that there was a statistically significant difference in OS (*p* = 0.01) between the two arms.^24^ Immune mechanisms such as antigen presentation‐related gene activation, increased immune lymphocyte infiltration, and PD‐L1 expression explain the effects of ICIs.^13^ For high‐risk patients with University of California, Los Angeles integrated Staging System classification, immune combined targeted therapy is the preferred strategy. We compared the effects of immunotherapy combined with TKIs versus TKIs alone in the two groups. Overall, the prognosis of patients with TKIs combined with ICIs was better than that of patients with TKIs alone. However, we did not subdivide the patients into different risk strata for further analysis, and subsequent analysis with an expanded sample size is still required.

Different histological components are also one of the factors affecting tumor prognosis. Rhabdoid and sarcomatoid differentiation can occur in any histological type of RCC, most commonly in clear cell RCC. A retrospective study by Janisch et al.^2^ showed that patients with mRCC‐S had more frequent clear cell histology (81.7%) findings, which is consistent with our cohorts. In our study, 11 of 12 patients (91.7%) with RCC‐S and all patients with RCC‐R had clear cell histologies. The proportion of rhabdoid and sarcomatoid differentiated tissue components also affects disease prognosis. Some studies have pointed out that when the sarcomatoid component is less than 25%, the effect after receiving TKI treatment is similar to that of ordinary RCC,^14^ and when the proportion of sarcomatoid differentiation is more than 25%, the prognosis is worse.^25^ In addition, patients with a sarcomatoid component of >30% of the primary tumor often have sarcomatoid differentiation in the metastases. Whether there are differences in the therapeutic effects of TKIs on patients with renal cancer with different subtypes and different ratios of rhabdoid and sarcomatoid differentiation components still needs further research.

Due to the retrospective nature of our study, which could have introduced an unadjustable selection bias, there were some limitations. In particular, the distribution of tumor stages in the patients was very uneven, especially the small number of T2 stage cases, which may have affected the analysis results. The number of patients still needs to be further expanded for multivariate regression analysis. Despite these constraints, the analysis provided the following results: (1) there may be differences in the efficacy of TKIs in mRCC‐R and mRCC‐S that need further investigation; (2) immunotherapeutic agents have a role in the prognosis of metastatic RCC.

## AUTHOR CONTRIBUTIONS

**Kun Wang:** Investigation (equal); methodology (equal); writing – original draft (equal); writing – review and editing (equal). **Pengqiang Duan:** Conceptualization (equal); software (equal); visualization (equal); writing – original draft (equal); writing – review and editing (equal). **Xusheng Chen:** Investigation (equal); project administration (equal); supervision (equal). **Qing Yang:** Formal analysis (equal); validation (equal). **Guowei Feng:** Investigation (equal); resources (equal). **Lei Diao:** Funding acquisition (equal); resources (equal); visualization (equal). **Zhenting Zhang:** Conceptualization (equal); resources (equal). **Xin Yao:** Project administration (equal); supervision (equal).

## CONFLICT OF INTEREST STATEMENT

The authors declare that there are no conflicts of interests.

## ETHICS APPROVAL STATEMENT

The study has been approved by the Medical Ethics Committee of Tianjin Cancer Hospital (approval no. bc2023044).

## Supporting information


Table S1.
Table S2.

## Data Availability

The data that support the findings of this study are available from the corresponding author upon reasonable request.
