# Supplementary material for: Comparison of tyrosine kinase inhibitors in the treatment of metastatic renal cell carcinoma with rhabdoid and sarcomatoid differentiations
Source: Cancer Med. 2023 Jun 16;12(13):14149–56. doi: 10.1002/cam4.6081 (PMC10358213; doi:10.1002/cam4.6081)
Supplement: Supplementary file 1 — Table S1. Table S2. [file CAM4-12-14149-s001.docx]

**Supplemental table 1**: Baseline characters of patients who has RCC-R and RCC-S.

|  | RCC-R(n=26) | RCC-S(n=44) | P value |
| --- | --- | --- | --- |
| Age, years | 58.38±8.55 | 59.55±9.55 | 0.601 |
| Man, n (%) | 19(73.1%) | 35(79.5%) | 0.367 |
| Tumor site(left), n (%) | 16(61.5%) | 23(52.3%) | 0.308 |
| Tumor size, cm | 7.43±2.18 | 6.72±2.59 | 0.249 |
| Tumor T stage, n (%) |  |  | 0.261 |
| T1+T2 | 6(23.1%) | 15(34.1%) |  |
| T3 | 20(76.9%) | 23(52.3%) |  |
| T4 | 0 | 6(13.6%) |  |

**Supplemental table 2**: Baseline characters of total population and included population.

|  | Total population  (n=111) | Included population  (n=23) | P value |
| --- | --- | --- | --- |
| Age, years | 58.77±9.55 | 58.35±6.87 | 0.839 |
| Man, n (%) | 86(77.5%) | 19(82.6%) | 0.790 |
| Tumor site(left), n (%) | 63(56.8%) | 12(52.2%) | 0.687 |
| Tumor size, cm | 7.36±3.38 | 7.57±2.83 | 0.778 |
| Tumor T stage n (%) |  |  | 0.189 |
| T1+T2 | 32(28.8%) | 10(43.5%) |  |
| T3 | 71(64.0%) | 10(43.5%) |  |
| T4 | 8(7.2%) | 3(13.0%) |  |
